# One more step in the study of children’s daily stress: The spillover effect as the transfer of tension in family and school environments

**DOI:** 10.3389/fpsyg.2022.909928

**Published:** 2022-12-07

**Authors:** Lidia Infante-Cañete, Lidia Arias-Calero, Agustin Wallace-Ruiz, Ana María Sánchez-Sánchez, Ángela Muñoz-Sánchez

**Affiliations:** ^1^Department of Developmental Psychology, University of Málaga, Málaga, Spain; ^2^Department of Psychobiology and Methodology of Behavioral Sciences, University of Málaga, Málaga, Spain

**Keywords:** spillover, daily stress, daily record, family, peers

## Abstract

**Introduction:**

The spillover effect is the psychological overflow due to daily stress in one context and the transfer of its consequences to another close environment. The aim is to explore the spillover effect in conflicts within the family, on the one hand, and school with peers on the other hand, as an inferred measure of daily stress according to the literature.

**Method:**

The study consisted of a sample of 208 6-year-old students and their families. A methodology based on daily report records was used, by means of two ad hoc checklists with simultaneous measurements, for 2 consecutive weeks and 3 academic years, for both family and school contexts. A repeated measures design, together with a nonparametric statistical data analysis with Friedman’s test and contrast measures, was used.

**Results:**

Daily stress shows significant differences in the family setting throughout the week (*χ* ^2^ = 32.44; *p* = 0.000) and at different times of the day (*χ* ^2^ = 29.65; *p* = 0.000). In the school setting, differences were found across the different days of the week (*χ* ^2^ = 36.96; *p* = 0.000). Spillover effect has been discovered between conflicts at home in the evening and conflicts at school. At the same time, conflicts at school are related to conflicts at home from Wednesday onward.

**Discussion:**

The results suggest further research on daily stress through the interrelation of the different contexts, as well as the impact that moments of conflict may have on the psychological and emotional development of the child.

## Introduction

The concept of spillover comes from areas of knowledge such as economics, engineering, physics, or ecology. It refers to those cases when something happening in one context directly affects a different context (Piotrkowski, 1979; Krpan et al., 2019). This very physical concept has been adapted to psychology (Crouter, 1984) and can be understood as the transfer of mood from one context to another due to excessive situational stress (Staines, 1980). More recently, Pakman (2006) defines the spillover effect as the psychological spillover that takes place when, after a temporary process of tension accumulation, it reaches such an intensity that the equilibrium is broken and the homeostatic mechanisms are saturated, experiencing an emotional overflow because of this cumulative process. The study of the spillover effect is therefore placed within the framework of stress and, more specifically, daily stress. Daily stress refers to the reaction—physical, psychological, and emotional—produced by a series of frustrating and irritating demands which occur daily when interacting with our environment (Kanner et al., 1981). Therefore, it is related to small events, problems, worries, and both high and low frequency setbacks, which are quite predictable and destabilize the physical-emotional wellbeing of the person (Seiffge-Krenke, 2007). The spillover effect implies an addition to the daily stress concept, as it brings in the export of those tensions produced by continuous events in one context to a totally different context, which can manifest themselves at the behavioral, psychological, or emotional level.

Different studies have addressed this transfer of tensions between interrelated contexts, as well as between those agents involved in the contexts. Some of these have focused on the work environment and how work demands affect family relationships (Crouter, 1984; Gerard et al., 2006; Flook and Fuligni, 2008), the way in which the quality of the couple’s interaction influences their children (Buckhalt et al., 2009; Nelson et al., 2009; Chung et al., 2011; Kouros et al., 2014), and how the household demands influence work outcomes (Crompton and Lyonette, 2006). Collectively, all these studies confirm the existence of the spillover effect. They also state that contagions between contexts or different agents in the same environment are interrelated, but they can be understood as independent concepts, and should be measured separately (Jacobs and Gerson, 2004; Hill, 2005; Ferrarini, 2006). In this sense, the ecological-systemic perspective (Minuchin, 1982; Bronfenbrenner, 2002) considers that the family microsystem is interdependent on a different microsystem—the school. In turn, both depend on the macrosystem where they are located, which is the residential area or neighborhood. According to Bronfenbrenner (2002), child development is subject to the influence of interconnected, network-like environments.

Both the family context through family member interaction and the school context through peer interaction are subject to multiple situations, of which a high percentage can generate daily stress. Children are not exempt from experiencing such daily stress, as well as the impact on their development and wellbeing (Trianes, 2002; Trianes and Escobar, 2009). Thus, different studies present the family and the school as the main stress-producing contexts (Phelan et al., 1994; De Anda et al., 2000). At the same time, they are essential for child development (Kaufman et al., 2020). In children, social relationships between peers and the conflicts derived from these relationships stand out as typical stressors. More specifically, Oros and Vogel (2005) point to fighting, being a bully, maintaining dominant and bossy behavior, and being bullied as behaviors related to this type of stressor. Being or feeling isolated from other schoolchildren is another stressor that has also been observed in this population (Salmivalli et al., 2000; Ortega and Monks, 2005). Several authors show that most school conflicts occur during recess, as it is a free environment to interact and relate with peers (Olweus, 1991; Rivers and Smith, 1994; Félix et al., 2008; Filella et al., 2016). Regarding the family environment, tensions within the family members are a negative factor for children’s development (Franz and Gross, 2001; El-Sheikh et al., 2006; Sandín, 2008; Buckhalt et al., 2009; Kouros et al., 2014). In particular, at early ages, they can become recipient subjects of parental tension spillover (Greenberger et al., 1994), but they can also be generators by hindering performance at work (Grzywacz et al., 2002; Grzywacz and Carlson, 2007) or parental couple life (Lawrence et al., 2010; Nelson et al., 2014). Consequently, the family’s daily routines and practices related to caregiving tasks such as feeding and dressing the kids, brushing their teeth, showering them, potty training them, or asking them to tidy up their things—usually observable activities repeated over time, which respond to physiological needs and are hardly modifiable (Wolin and Bennet, 1984)—would theoretically be linked to promoting the children’s wellbeing and health, but may entail both a protective and a destructive value (Palacios and Andrade, 2008; Fiese and Winter, 2010), since they are not exempt from generating conflicts and tensions within the couple or between the couple and the children (Sears et al., 2016). Therefore, dyadic as well as triadic interactions are relevant (Stroud et al., 2011).

Currently, there are barely any studies on the spillover effect at 6 to 8 years, since the literature reviewed in the population under 18 years of age focused either on early childhood or on adolescence and pre-adolescence (Salamon et al., 2011; Stroud et al., 2011; Martin et al., 2017; Davies et al., 2018; Pu and Rodríguez, 2021; Mastrotheodoros et al., 2022). This age range is crucial because from the age of 6, children have to face important changes and events such as the transition from Early Childhood Education to Primary School (Rimm-Kaufman and Pianta, 2000; Schulting et al., 2005; Bulkeley and Fabian, 2006; Fabian and Dunlop, 2006; Dockett and Perry, 2007). A higher degree of autonomy and responsibility is demanded from them by two institutions which are not mutually consistent in their requirements (Oliva and Palacios, 2000; Bronfenbrenner, 2002; Enns et al., 2018), in addition to greater academic demands mediated by stress and anxiety (Ramírez et al., 2016) while they begin to be aware of their emotions and it is a prime age to work on emotional management, due to its impact on learning and academic success (Graham et al., 1984; Palacios and Hidalgo, 1999; Ortiz, 2001; Herrera et al., 2004; Soldevila et al., 2007; Denham et al., 2016; Enns et al., 2018). According to Kinkead-Clark (2015), an essential element with an impact on academic transitions is the quality of educational and parenting practices in the years prior to Primary Education. Moreover, during these early years of Primary Education, a strong and greater socialization among peers is expected, and the conflict resolution strategies used in Early Childhood Education give way to more complex strategies. Therefore, the first cycle of Primary Education corresponds to the age interval where more conflicts occur (Soldevila et al., 2007; Sidorowicz et al., 2009; Filella et al., 2016).

Having made this point, it is worth tracing how conflicts appearing in the school context when interacting with peers produce a transfer to the family context (and vice versa) in children (Lehman and Repetti, 2007; Nelson et al., 2009; Chung et al., 2011; Du et al., 2018), since there is still not much knowledge about the way this mutual relationship works (Kaufman et al., 2020). A precedent for this can be found in Flook and Fuligni (2008), who examined this spillover effect in adolescents, reporting results that showed a bidirectional influence between daily family spillover and conflictive school relationships. In particular, difficulties in school social relations seem to be closely related to family difficulties, as well as to the child’s school adjustment (Repetti et al., 2002). Thus, several studies have shown how behavioral problems in childhood predict parental stress which, in turn, predicts the appearance of behavioral problems in children. It shows that, if the effects are bidirectional, it is possible to reduce behavioral problems in case a decrease in parental stress is achieved (Baker et al., 2005; Crnic et al., 2005; Mackler et al., 2015). The quality of the relationship with peers is also important in relation to the psychological adjustment of children, especially when it is not satisfactory and/or they suffer rejection (Trianes, 2002).

In sum, this study starts from the need to analyze the spillover effect between the family context (defined through the conflictive interactions occurring in the daily parenting activities that parents have with their children) and the peer context through the conflictive interactions that children have with their classroom peers, through the daily report. The daily report methodology allows recording—on a daily basis and in detail—how short-term tensions are transmitted between the parenting process carried out by parents and their children’s relationships with their peers (Almeida, 2004; Chung et al., 2011). Daily reports or records are useful when the aim is to catch or capture, whatever the context, the set of micro-processes of tensional spillover at the particular moment in which they take place. Thus, it is possible to analyze the appearance of daily tensions in the family system and the consequent effects on the relationship between children and their peers. On that account, the daily record allows detecting specific episodes of tension both at home and at school, in a short interval of time, from the moment they take place in a given context. This methodology has already been used in recent studies on the spillover effect between parents and adolescents and preadolescents, showing satisfactory results. The daily report has been used in studies such as those of Lehman and Repetti (2007), who evaluated for 5 consecutive days the events that took place at school and at home, or in the work of Flook and Fuligni (2008), in which the evaluation was conducted with adolescents during 2 consecutive weeks. Sherrill et al. (2017) is also another example in the use of daily records. All of these studies report a basal functioning or behavioral pattern where behavioral differences respond to an objective, non-arbitrary fact across contexts (Lehman and Repetti, 2007; Flook and Fuligni, 2008; Sherrill et al., 2017).

This study has the following objectives:

1. To identify the existence of a possible pattern of conflict behaviors in each context throughout the week. Studies focusing on the spillover effect report a basal functioning in both preadolescents (Sherrill et al., 2017) and adolescents (Lehman and Repetti, 2007; Flook and Fuligni, 2008), where conflict behaviors are repeated and show some kind of interrelation. The aim is to capture the existence of this basal rhythm, as objective and non-arbitrary data, in a smaller population than that studied so far in the literature. In this respect, the demands and requirements that the 6–8 year-old children must face are the change from Early Childhood Education to Primary School, which requires a greater degree of autonomy and responsibility, more academic demands, and a deeper awareness of their emotions, as mentioned above. Therefore, from 6 to 8 year, the child is in the same educational cycle and developmental stage (Ballesteros and García, 2001; Coll et al., 2014) that may provide certain stability in the different contexts, so that it may favor the attainment of the behavioral pattern that supposes that basal rhythm (Gerard et al., 2006).
2. To estimate the prevalence of both parenting conflicts within the family context and peer conflicts within the school context, as measures of context-specific tensions. Studies such as those of Palacios and Andrade (2008); Trianes et al. (2009); Filella et al. (2016) on the school setting, as well as those of Stroud et al. (2011) or Sears et al. (2016) on the family setting, point to behaviors related to stressors. In this sense, finding comparable conflicts in the scientific literature means that our conclusions can be generalizable and replicable, since the sample could not be considered specific or biased by any particular aspect.
3. To analyze the bidirectional relationship between parental conflicts on the subject of children upbringing, and conflicts involving these children and their peers at school, for 3 years. Studies with populations larger than the study sample have discovered a spillover effect in different moments (Lehman and Repetti, 2007; Flook and Fuligni, 2008; Chung et al., 2011; Bai et al., 2017; Sherrill et al., 2017), and it is expected to be able to specify the existing relationship between conflicts in different contexts.

In relation to hypotheses, the following is expected:

*Hypothesis 1*: Parenting conflicts occurring early in the morning will negatively affect children throughout the day at three points: in the development of interactions with peers at school, in the development of parenting activities in the afternoon, and in the development of parenting activities in the evening hours.

*Hypothesis 2*: Conflict situations occurring in the family home follow a characteristic pattern throughout the week, which is systematic in each year evaluated.

*Hypothesis 3*: Prevalence data on school and family conflicts will be compatible with those found in the scientific literature.

*Hypothesis 4*: The interaction conflicts between peers in the school context will have a negative effect on daily parenting tasks twice a day: in the afternoon and in the evening.

*Hypothesis 5*: The spillover effect between family and school conflict situations can be bidirectional.

## Materials and methods

### Participants

A total of 208 students (104 boys and 104 girls) participated, together with their respective parents. The starting age for schoolchildren is 6 years old. The sample came from four first-year primary school classes from two public schools in the province of Malaga. These were located in a medium-high socioeconomic level area.

### Instruments

#### Daily report on interpersonal conflicts with peers in the school context

It is an *ad hoc* self-report assessing the micro-processes of interpersonal conflicts as they occur at school (see Appendix 1). Its content is extracted from empirical studies and current theoretical reviews related to the area of interpersonal conflicts in childhood (Salmivalli et al., 2000; Oros and Vogel, 2005; Ortega and Monks, 2005). It consists of 13 items in a Yes/No response format. In addition, the positive answer is graduated according to frequency: once, 2–4 times, 5 o or more times. This report yields two scores—the first one is the total score of the report and ranges from 0 to 13 points, whereas the second one is the number of conflicts the student has been involved in that morning. The second score is the frequency of conflictive events the student has been caught up in during the morning, and ranges from 0 to 39 points. In this study, the total score has been used.

#### Daily report on parenting conflicts between parents and their children

This is an instrument which has been developed *ad hoc*, in order to assess the micro-processes of interpersonal conflicts when they occur in the daily routine between parents and children. Specific and updated literature related to stressful family interpersonal conflicts was consulted for its elaboration (Wolin and Bennet, 1984; Almeida, 2004; Palacios and Andrade, 2008; Fiese and Winter, 2010; Chung et al., 2011; Stroud et al., 2011; Sears et al., 2016; Sherrill et al., 2017); see Appendix 2. For its design, specific and updated literature related to the area of stressful family interpersonal conflicts was consulted. The daily report focuses on the daily tasks. In each one, it is evaluated whether each child performs the main task of that moment without posing any conflict or, on the contrary, presents resistance behaviors, such as being sad, angry, or uncooperative, demanding attention, throwing tantrums, being aggressive, or disobeying. This report yields three scores that refer to morning parenting conflicts (from 0 to 28 points), afternoon parenting conflicts (from 0 to 27 points), and evening parenting conflicts (from 0 to 18). The score is the number of parenting conflicts where the child has been involved. The report also yields a total score ranging from 0 to 73 points—this score corresponds to the number of parenting conflicts throughout the day. It is important to note that, in these scores, items that had a positive wording were excluded, as they did not show conflict. The items referring to “Other problem” offered the families the possibility of describing another situation that differed from those considered.

### Procedure

After the selection of the educational centers that showed interest in participating in the research project *“Spillover between parent–child and child-peer*. *Transmission of bidirectional tensions between parent–child (6–8 years old) and child-peer interactions,”* with project code SEJ2011-7225, the pertinent permissions were requested from the authorities of the Education Department in the province of Malaga, the school board, and the teaching staff of each of the participating centers. Informative meetings were held with both teachers and families with the aim of informing them about the aims of the research. Besides, the signing of a collaboration agreement was asked—specific aspects such as space, timetable, and procedure were specified there. At the same time, the research team was committed to process the data confidentially, make them available to the center’s guidance department and the parents, contribute to the training of parents, and collaborate in other initiatives undertaken by the center during the development of the project. Prior to this study, a pilot study was carried out to test the instruments and data collection.

The *Daily reports on interpersonal conflicts with peers in the school context* were completed individually by means of personal interviews from Monday to Friday during school hours, in the time slot after recess. The instruments were administered by four researchers who told the children about the voluntary nature of participation.

The *Daily Parenting Conflict Reports* were completed individually by the primary caregiver during the last hour of the day in the same data collection period for their children. The report was collected the morning after its completion in a sealed envelope by the tutor responsible for the class under research.

This procedure was approved by the Ethics Committee of the University of Malaga, within the Excellence Project SEJ-7226, funded by the Junta de Andalucía.

### Data analysis

Based on a longitudinal design, the descriptive data of all the study variables and the normality check of their distribution are presented in the first place. Secondly, in order to test the spillover effect, different statistical analyses were performed—Spearman’s correlation and Friedman’s test. The statistical software package for Social Science (v25) was used for the sake of data analysis.

## Results

The analysis of descriptive statistics shows that the selected variables do not follow a normal distribution, which implies the use of non-parametric statistical tests. Previously, a Mann–Whitney *U* test showed that there were no differences between sexes (for morning, afternoon, and evening respectively, *U* = 3807.5, *p* = 0.156; *U* = 3732.5, *p* = 0.361; *U* = 3734.0, *p* = 0.126) or between schools (for morning, afternoon, and evening, respectively, *U* = 3461.5, *p* = 0.117; *U* = 3376.5, *p* = 0.102; *U* = 3418.0, *p* = 0.108). The descriptive statistics of the data (see Table 1) show that the number of conflicts collected during the three consecutive academic years of the research was not very high.

**Table 1:** Descriptive statistics.

	Average	Median	Mode	Standard deviation
Morning	1.418	0.6	0	2.002
Afternoon	1.440	1	0	1.762
Evening	0.944	0	0	1.440

It was also explored which conflict situations were most likely to occur in each context by means of a statistical analysis of frequencies (Table 2). The most common morning conflicts in the family environment are closely related to each other—these fundamentally correspond to four items associated with the refusal to do chores (“S/he does not want to get up,” “S/he does not cooperate with getting washed up,” “S/he does not cooperate when getting dressed” and “S/he does not eat breakfast”). In the afternoon conflicts, something similar happens—there are four items with a higher percentage of occurrence which are associated with aggressive behaviors, expression of negative emotions, and denial of the demand (“S/he appears angry about something,” “S/he disobeys in a particular situation,” “S/he fights with friends or siblings,” “S/he does not want to take care of his/her personal hygiene”). Finally, in relation to evening conflicts, the same trend continues, with the most prominent items being those related to the expression of sadness and lack of collaboration in the task (“S/he appears sad at dinner,” “S/he appears sad at bedtime,” “S/he does not cooperate at dinner” and “S/he does not cooperate when going to bed”). This table includes those potentially conflictive situations whose percentage exceeds 5%.

**Table 2:** Frequency distribution of items in the family conflict daily record.

Morning conflicts item	Percentage	Cumulative percentage
S/he appears cranky and demanding	6.56	40.91
S/he does not cooperate at breakfast	7.63	48.54
S/he does not eat breakfast	7.70	56.24
S/he does not want to get up	13.40	69.64
S/he does not cooperate in getting washed up	13.40	83.04
S/he does not cooperate when getting dressed	16.96	100
**Afternoon Conflicts Item**	**Percentage**	**Cumulative percentage**
S/he throws tantrums over food	6.70	31.33
S/he appears cranky and demanding	9.81	41.14
S/he appears angry about something	10.08	51.22
S/he does not want to take care of their personal hygiene	10.76	61.98
S/he disobeys in a particular situation	18.27	80.24
S/he fights with friends and/or siblings	19.76	100
**Evening conflicts item**	**Percentage**	**Cumulative percentage**
Another problem	5.35	20.93
S/he appears angry because s/he does not want to go to sleep	5.69	26.62
S/he appears cranky and demanding	9.44	36.06
S/he throws tantrums in order not to go to sleep	9.67	45.73
S/he does not want to eat their meal	10.35	56.09
S/he appears sad about something	11.26	67.35
S/he eats their meal without a fuss	14.68	82.03
S/he appears sad	17.97	100

On the other hand, the most common conflicts in the school environment also turned out to be mainly four. These situations account for more than 50% of the total (see Table 3), and can be potentially frustrating, since most of them (except for the item “You have been picked on and/or insulted”) are not based so much on direct aggression, but rather on the perception of a conflict in the face of a refusal or reproach from others (“You could not do what you wanted because others decided to do something else,” “They told you to calm down and be quiet,” “You could not borrow what you wanted”).

**Table 3:** Frequency distribution of items in the daily record of school conflicts.

School conflicts item	Percentage	Cumulative percentage
Have you had a fight with another child?	3.17	3.17
You have been accused for no reason	5.07	8.24
Someone did not want to be your friend	5.23	13.47
Have you ever had a confrontation with another child without getting into a fight?	5.67	19.14
You have imposed your will on other children	5.96	25.10
You have been attacked by another child (without having started the fight yourself)	6.44	31.54
You have been pushed (stepped on, any covert aggression)	6.98	38.51
You have not been allowed to play	7.36	45.87
You have gotten scolded for no reason	7.75	53.62
You have been told to calm down, to stay still	8.88	62.50
You have been unable to borrow something you wanted	8.93	71.43
They have picked on you (you have been insulted)	13.33	84.77
You have not been able to do what you wanted to do because other children have decided to do something else	15.23	100

After having carried out this step, it was tested whether there parenting conflicts between parents and children throughout the day (morning, afternoon, and evening) during all days of the week, in the 3 years of the study. Statistically significant correlations were found between morning-afternoon [*r* = 0.434^**^; *p* < 0.001], morning–evening [*r* = 0.392^**^; *p* < 0.001], and afternoon-evening [*r* = 0.416^**^; *p* < 0.001]. These correlations were the strongest between the times of day closest to each other.

Friedman’s test was performed to check if there were statistically significant differences in the number of morning, afternoon, and evening conflicts. Upon finding statistically significant differences, several Wilcoxon *t*-tests with Bonferroni correction were carried out to see between which groups there were differences. The findings show that more conflicts happened in the morning than in the evening, and more conflicts occurred in the afternoon than in the evening. The results of the effect size calculation indicate that the effect size is greater between morning and evening conflicts (Table 4).

**Table 4:** Results of Friedman’s test and multiple *a posteriori* contrasts of conflicts at home.

Comparison mornings: Afternoons—evenings							
Friedman	*χ* ^2^	*p*					
MC-AC-EC	29.65	0.000	^**^				
T Wilcoxon	*M*	SD	*M*	SD	*T*	*p*	
Morning conflict–afternoon conflict	6.935	8.445	6.372	6.879	5442.50	0.783	
Morning conflict–evening conflict	6.935	8.445	4.616	5.998	3337.50	0.000^**^	*r* = 0.24
Afternoon conflict–evening conflict	6.372	6.879	4.616	5.998	3209.50	0.000^**^	*r* = 0.22

*p < 0.05; **p < 0.01.

It was also tested whether the amounts of conflicts during the days of the week were similar or differed from each other. Using the same statistical tests as in the previous analysis, it was found that Monday is the day with the highest amount of morning conflicts with respect to the rest of the days of the week (Table 5). The results of the effect size calculation indicate that the effect size increases throughout the week.

**Table 5:** Results of Friedman’s test and multiple *a posteriori* contrasts of conflicts at home in the morning during weekdays.

Comparison parents days of the week morning							
Friedman	*χ* ^2^	*p*					
M-T-W-Th-F	32.44	0.000	^**^				
T Wilcoxon	*M*	SD	*M*	SD	*T*	*p*	
Monday–Tuesday	1.887	2.313	1.432	1.904	1684.00	0.001^**^	*r*=0.18
Monday–Wednesday	1.887	2.313	1.421	2.206	1580.00	0.000^**^	*r*=0.21
Monday–Thursday	1.887	2.313	1.258	1.785	1652.00	0.000^**^	*r*=0.22
Monday–Friday	1.887	2.313	1.202	1.866	1305.50	0.000^**^	*r*=0.25
Tuesday–Wednesday	1.432	1.904	1.421	2.206	2148.00	0.505	
Tuesday–Thursday	1.432	1.904	1.258	1.785	2086.50	0.066	
Tuesday–Friday	1.432	1.904	1.202	1.866	1567.00	0.011	
Wednesday–Thursday	1.421	2.206	1.258	1.785	1762.00	0.405	
Wednesday–Friday	1.421	2.206	1.202	1.866	1564.00	0.096	
Thursday–Friday	1.258	1.785	1.202	1.866	1816.50	0.436	

*p < 0.05; **p < 0.01.

During the afternoons, there are more conflicts on Mondays than on Thursdays and Fridays, and there are more conflicts on Tuesdays and Wednesdays than on Fridays. In this case, the effect size is very similar, although the same trend as in the previous analysis could be observed (Table 6).

**Table 6:** Results of Friedman’s test and multiple *a posteriori* contrasts of home conflicts in the evenings during weekdays.

Comparison parents weekdays evenings							
Friedman	*χ* ^2^	*p*					
M-T-W-Th-F	21.204	0.000	^**^				
T Wilcoxon	M	SD	M	SD	T	*p*	
Monday–Tuesday	1.790	2.337	1.601	1.716	1241.00	0.424	
Monday–Wednesday	1.790	2.337	1.587	1.725	1770.50	0.533	
Monday–Thursday	1.790	2.337	1.300	1.711	1211.00	0.004^**^	*r* =0.17
Monday–Friday	1.790	2.337	1.167	1.515	1256.00	0.002^**^	*r* =0.18
Tuesday–Wednesday	1.601	1.716	1.587	1.725	1885.50	0.756	
Tuesday–Thursday	1.601	1.716	1.300	1.711	1337.50	0.042	
Tuesday–Friday	1.601	1.716	1.167	1.515	1335.50	0.005^**^	*r* =0.16
Wednesday–Thursday	1.587	1.725	1.300	1.711	1295.50	0.036	
Wednesday–Friday	1.587	1.725	1.167	1.515	1023.00	0.001^**^	*r* =0.19
Thursday–Friday	1.300	1.711	1.167	1.515	1525.00	0.235	

*p < 0.05; **p < 0.01.

During the evenings, there were no differences in the number of conflicts throughout the week [*χ*^2^ = 7.001; *p* > 0.05].

Regarding interpersonal conflicts with peers in the school context, it was checked whether they changed throughout the week, finding that there were more conflicts on Mondays than on Thursdays and Fridays, and that the number of conflicts was higher on Tuesdays, Wednesdays, and Thursdays than on Fridays. The effect size is larger the farther apart the days of the week are, i.e., results like the previous analyses are obtained (Table 7).

**Table 7:** Results of Friedman’s test and multiple *a posteriori* contrasts of interpersonal conflicts with peers in the school setting.

Equal days of the week comparison							
Friedman	*χ* ^2^	*p*					
M-T-W-Th-F	36.96	0.000	^**^				
T Wilcoxon	*M*	SD	*M*	SD	*T*	*p*	
Monday–Tuesday	8.679	6.538	7.989	6.466	5889.50	0.072	
Monday–Wednesday	8.679	6.538	7.978	6.605	5886.50	0.071	
Monday–Thursday	8.679	6.538	7.527	6.333	5014.50	0.003^**^	*r* =0.16
Monday–Friday	8.679	6.538	6.592	5.964	3643.00	0.000^**^	*r* =0.28
Tuesday–Wednesday	7.989	6.466	7.978	6.605	6525.50	0.993	
Tuesday–Thursday	7.989	6.466	7.527	6.333	5672.00	0.119	
Tuesday–Friday	7.989	6.466	6.592	5.964	4055.50	0.000^**^	*r* =0.21
Wednesday–Thursday	7.978	6.605	7.527	6.333	5619.00	0.201	
Wednesday–Friday	7.978	6.605	6.592	5.964	4437.00	0.000^**^	*r* =0.18
Thursday–Friday	7.527	6.333	6.592	5.964	4518.00	0.003^**^	*r* =0.15

*p < 0.05; **p < 0.01.

Through a previous Spearman correlation analysis, it was found that evening conflicts were related both to morning conflicts the following day [*r* = 0.403^**^; *p* < 0.001], and to interpersonal conflicts with peers in the school setting [*r* = 0.175^*^; *p* < 0.05].

Also, it was checked whether there was a relationship between interpersonal conflicts at school and conflicts at home in the afternoon and evening during the weekdays, in the 3 years of the study. No relationship was found between school conflicts and family and interpersonal conflicts in the afternoon, but significant correlations were observed from Wednesday onward between school and family conflicts in the evening hours (Table 8).

**Table 8:** Results of Spearman’s correlations of interpersonal conflicts and conflicts at home throughout the week.

	Afternoon conflict	Evening conflict
Interpersonal Monday	−0.17	−0.04
Interpersonal Tuesday	0.12	0.18
Interpersonal Wednesday	0.02	0.18^*^
Interpersonal Thursday	0.02	0.32^**^
Interpersonal Friday	0.02	0.39^**^

*p < 0.05; **p < 0.01.

## Discussion

The use of the concept of spillover linked to the field of psychology is fairly recent (Pakman, 2006) and is connected to the concept of daily stress (Seiffge-Krenke, 2007). The term “spillover” completes the effect that daily stress has on people’s lives insofar as it contemplates the transfer of its negative effect to another context, so that one area of life can impact another one. This results in an amplification of responses that may be conflicting, for example, between the family and school context (Repetti and Wang, 2017).

This study delves into the idea of how the conflicts occurring in one context, family or school, are related to the daily difficulties experienced in the other context. In this sense, this study aims to detect the pattern of the spillover effect both on a single day and throughout the week, as well as to analyze the bidirectional relationship between the conflicts experienced by parents in carrying out parenting activities with their children, and conflicts experienced by these children and their peers at school, over a three-year study period. In addition, the most prevalent conflicts in each of the contexts evaluated in the selected sample have been described. Based upon a repeated measures design, nonparametric tests have been used to demonstrate different relationships between the data.

The study findings indicate—in relation to the first hypotheses—that a repetitive pattern is observed throughout the 3 years of the study, with Monday being the day with the highest level of conflict in both contexts. In turn, Monday’s potential for conflict also shows its own pattern, with the first moments of the day registering the highest incidence. Friday is the day with the lowest number of conflicts (especially in the afternoon), so the likelihood of conflict decreases as the week progresses and the weekend approaches. Finally, it should be noted that, in general, mornings are more eventful than evenings. Thus, the hypothesis is corroborated, and the first objective of the research is fulfilled—this is very important, since there are no references in the scientific literature about the pattern followed by the family-school spillover effect, so this finding is of great relevance so as to know the specific moments and days which are more prone to spillover.

In relation to the second hypothesis, regarding the prevalence of problematic situations in each environment, although there is not a large number of conflicts in the study sample, most of those happening in the family context have to do with refusal behaviors on the part of the child to perform tasks, as well as with behaviors of expressing negative emotions in performing the requested behavior. Conflicts with parents as a result of living together have also been considered as a potential area of stress prevalent in reviews such as that of Trianes et al. (2009) Other authors such as Palacios and Andrade (2008) point out that parental practices within the family are linked to positive or negative behavioral responses—at the same time, they are an essential element for understanding problematic behaviors in both childhood and adolescence. The results of this study are consistent with this approach. In the school context, conflicts are more related to non-validation of the child’s behavior or direct denial of a child’s request by others. This is consistent with the scientific literature reviewed, which includes incidents with classmates, peer rejection, and other problems related to interaction such as feelings of loneliness as frequent sources of stress in children between 6 and 12 years of age (Salmivalli et al., 2000; Ortega and Monks, 2005). However, our results differ from those of Filella et al. (2016), who conclude that physical and verbal aggression were the most frequent types in the study.

For hypothesis #3 the data reveal a significant correlation in conflicts originating at home at different times of the day—the closer they are to each other, the greater the correlation. This means that a conflict happening in the morning is related to the possibility of a conflict occurring in the afternoon—in fact, the morning is the time of the day when conflict is most likely to take place, and the evening is the time of the day when it is least likely to occur. On the other hand, a significant relationship was also observed between the conflicts occurring at home in the evening and the conflicts happening the following morning, both in the family and school contexts (hypothesis #4). Therefore, a spillover effect is observed within the family environment during the different times of the day—the following day is included in this spillover effect—, thus observing a spillover effect between the events occurring at the end of the day both in the family and the school contexts, adding information to the pattern demonstrated for the first objective. These findings are consistent with the existing scientific literature (Greenberger et al., 1994; Cummings and Davies, 2010; Fiese and Winter, 2010) suggesting that parenting tasks can be causal factors of child spillover which affect their development and manifest themselves in the school context in the form of conflicts with peers.

In relation to hypothesis #5, which states that peer interaction conflicts occurring in the school context will negatively affect daily parenting tasks at two times of the day—in afternoon parenting activities and in the development of such tasks in the evening, the spillover effect is only captured from Wednesday onward, through the connection between peer-peer conflicts and evening conflicts at home. This behavioral pattern that shows the spillover effect from Wednesday onward refers to the accumulation process described by Pakman (2006), who states that after an accumulation of tensions there is a rupture of equilibrium, leading to overflow and transference. In the same sense, and described at a more general level, the study by Kaufman et al. (2020) states that tension suffering due to victimization situations with peers is associated with conflicts in the parental-filial relationship.

Finally, the third objective was to analyze a possible bidirectional relationship between conflicts occurring at home and at school, respectively.

The results obtained allow us to state that this bidirectionality exists in the transfer of tensions (sixth hypothesis), although this spillover effect is linked to particular moments and events. The spillover effect between the family and school contexts is reflected daily through the relationship between evening events at home and peer-peer tensions in the school context the following day. Nevertheless, the spillover effect between the school and family contexts is only captured from Wednesday onward and through the relationship between interpersonal conflicts between peers and evening conflicts at home. Consequently, the findings of this study are in line with the research of Repetti et al. (2002), which shows how school conflicts have their origin in family conflicts happening at home. It is also related to other publications where a spillover effect between the two contexts is identified through daily log reports (Bai et al., 2017), where positive same-day correlations were reported, and other work where the described bidirectional effect could be ascertained (Crnic et al., 2005; Flook and Fuligni, 2008; Mackler et al., 2015), as a “mirror image” according to Krpan et al. (2019). However, the results found between the school and the family contexts suggest a spillover effect due to a cumulative process of stress (Pakman, 2006)—in this case, school stress, as it was pointed out in the above paragraph. Therefore, the fact that the spillover effect does not take place during the first days of the week, but by the middle of the week, suggests that weekends could be a protective factor against it. Still, this possible relationship remains for further study.

When studying the spillover effect through correlational analysis, statistically significant associations do not always occur at all recorded and evaluated times, but only at particular moments, and the added fact that it is observed for 3 years in a row means that there is a cumulative effect of stress experiences. It should be noted that the longitudinal nature of the study—together with the data collection by daily recording during the different weeks—has undoubtedly allowed to capture the micro-relationships existing between the different moments (Chung et al., 2011; Sears et al., 2016; Sherrill et al., 2017). In other studies, based on questionnaires, it is not possible to detect this cumulative process, but the daily record method allows to do so. As Galizzi and Whitmarsh (2019) indicate, the longitudinal methodology does not identify causality, but it does quantify the strength of the relationships between the measured behaviors, providing a standardized measure.

The *main finding* of this study refers to the description of the spillover effect between the different moments of parenting within the family context and the spillover effect from the family context to the school context and vice versa at 6–9 years of age. In addition, a characteristic behavioral pattern has been revealed over time—where Mondays are the most difficult days in family living—that morning conflicts are related to later conflicts, and that conflicts at home are related with one another. Evenings are usually the most peaceful time of the day at home, and Fridays are the calmest days of the week. Evening conflicts at home are related to conflicts at school and conflicts at school from Wednesday onward are connected to conflicts at home due to a possible cumulative factor in relation to daily conflicts.

The *strengths* of this study are based on the fact that empirical evidence of the spillover effect is still poorly documented today, and most research addresses it cross-sectionally through questionnaires measuring this phenomenon in an *ad hoc* manner. The spillover effect requires knowledge of the temporal intertwining of tensions and behaviors in a longitudinal design. These are day-by-day studies with a longitudinal approach of small amplitude in terms of the temporal sequence studied with the daily report methodology. As mentioned, in the case of young children, there are barely any studies that investigate spillover in relation to the family and peer systems, since the few studies that exist do so with adolescents, whose chronological stage is completely different. Finally, the methodology based on the daily report, despite its limitations in this study, proves to be a versatile method capable of capturing the course of time.

## Limitations and future research

Although the spillover effect has been demonstrated in different times and contexts, this study has certain limitations. One of them was the low incidence of conflicting events obtained with the sample. One possible explanation may be the sociocultural context from which the families come and where the schools are located. This limitation can also be observed in other research such as that of Bai et al. (2017), who report little or few incidents. Another limitation is that the family socioeconomic level has not been specifically evaluated, since this variable was not considered relevant for the study when considering the conclusions of Flook and Fuligni (2008) and Nelson et al. (2009), where they did measure this variable, and it was determined that socioeconomic level does not influence *per se* the spillover effect. Despite these conclusions, it would be convenient to take this into account in future work for greater objectivity.

In future research, it would be interesting to use samples with different conflict incidence rates, and to carry out comparative studies through the relationship between the different development contexts among various cultural environments. On the other hand, it is essential to continue research with the development of daily recording instruments that facilitate efficient data collection and meet the different criteria established according to criterion and construct validity. This study has described for the first time a behavioral pattern of related conflict behaviors, and it would be interesting to study how variables such as parenting styles, social skills, or coping strategies influence this relationship and pattern.

### Implications for psycho-educational intervention

Stress linked to a particular life event has a great impact on the individual’s development and on how they relate to their context during a given time—however, everyday stress can have a greater impact on the development of the child or adolescent (Seiffge-Krenke, 2000) since high levels of everyday stress are associated with significant negative consequences of emotional maladjustment and psychopathology (Seiffge-Krenke, 2000; Jose and Ratcliffe, 2004) that are prolonged over time. Consequently, the use of a more global theoretical and methodological perspective where the relationship of several interrelated mycosystems is taken into account can help to learn even more about the stressors with respect to such maladjustment, as well as the type of psychopathology which is more prone to situations and/or specific moments potentially susceptible to conflict, and the intrafamilial bonds affecting the education of children in this age group.

The psychoeducational intervention focused from the spillover perspective on daily stress should contemplate the behavioral pattern described here, as well as the cumulative effect of stressors, which give rise to the spillover of their negative effects. The aim of a psychoeducational intervention should be to reduce daily stressful events by promoting competent behaviors both in the family environment (through appropriate educational styles) and in the school environment, through socioemotional education. Reducing stressors, as well as having appropriate coping strategies, may hamper the cumulative process defining the spillover effect between contexts, thus increasing the individual’s well-being. The daily events occurring in the school environment do not produce an overload of stresses on their own, but it is their accumulation over the course of the days that produces a behavioral imbalance in the child, which is transferred to the family, with respect to those days when the spillover effect does not take place.

## Conclusion

Stress—both in adult life and in childhood—tends to be related to specific situations, but the findings of this study refer to the interrelation of contexts (Bronfenbrenner, 2002) and the way reactions produced by small events, problems, worries, and setbacks of high frequency, low intensity and high predictability (daily stress) can be transferred from a particular context to a completely different one, and can manifest themselves at the behavioral level through conflicts in the environment (spillover effect).

In this sense, this research has shown that the spillover effect is present from a very early age, with bidirectionality between family and school contexts, although this spillover effect is linked to particular moments and events. The data also provide a characteristic behavioral pattern indicating the days of greatest conflict and reflecting the process of accumulation that occurs during the week. On the other hand, it points out the usefulness of the longitudinal cut study and the use of reports as the most plausible way to know better the daily experiences lived according to the daily stress in the identification of key moments. Finally, it shows that, if the sources of stress are known—together with the particular moments of conflicts, the stress accumulation process, and how the behavioral transfer of its consequences from one environment to another—it will be easier to prevent and provide coping and stress control strategies to both families and their children. This reduces the adverse effects and contributes to both the psychological and emotional well-being of the person.

## Data availability statement

The original contributions presented in the study are included in the article/Supplementary material, further inquiries can be directed to the corresponding author.

## Ethics statement

The studies involving human participants were reviewed and approved by Universidad de Málaga. Written informed consent to participate in this study was provided by the participants’ legal guardian/next of kin.

## Author contributions

LI-C, LA-C, AS-S, and AM-S contributed to the conception and design of the study. AW-R organized the database and performed the statistical analysis. LI-C, LA-C, and AW-R wrote the manuscript. All authors contributed to the article and approved the submitted version.

## Funding

This work has received funding from the Excellence Project by the Junta de Andalucía in 2011–2013: Spillover between parent–child and child–peer conflicts. Transmission of bidirectional tensions between parent–child (6–8 years old) and child-peer daily interactions at school. Consequences for school performance, parental educational strategies, stress in parents and children, and psychopathology. Project code: SEJ-7226.

## Conflict of interest

The authors declare that the research was conducted in the absence of any commercial or financial relationships that could be construed as a potential conflict of interest.

## Publisher’s note

All claims expressed in this article are solely those of the authors and do not necessarily represent those of their affiliated organizations, or those of the publisher, the editors and the reviewers. Any product that may be evaluated in this article, or claim that may be made by its manufacturer, is not guaranteed or endorsed by the publisher.
