# Supplementary material for: One more step in the study of children’s daily stress: The spillover effect as the transfer of tension in family and school environments
Source: Front Psychol. 2022 Dec 7;13:909928. doi: 10.3389/fpsyg.2022.909928 (PMC9768336; doi:10.3389/fpsyg.2022.909928)
Supplement: Supplementary file 1 [file Data_Sheet_1.docx]

Supplementary Material

# Appendices

Appendix 1. ***Daily report on interpersonal conflicts at recess***

|  | First and last name:  Date:___________________________ | YES  How many times? | | | NO | What did you do? |
| --- | --- | --- | --- | --- | --- | --- |
|  |  | 1 | 2-4 | 5+ |  |  |
| 1 | Have you had a fight with another child? |  |  |  |  |  |
| 2 | You have been attacked by another child (without having started the fight yourself) |  |  |  |  |  |
| 3 | You have imposed your will on other children |  |  |  |  |  |
| 4 | You have not been allowed to play |  |  |  |  |  |
| 5 | Someone did not want to be your friend |  |  |  |  |  |
| 6 | You have been accused for no reason |  |  |  |  |  |
| 7 | You have gotten scolded for no reason |  |  |  |  |  |
| 8 | They have picked on you (you have been insulted) |  |  |  |  |  |
| 9 | You have been pushed (stepped on, any covert aggression) |  |  |  |  |  |
| 10 | You have not been able to do what you wanted to do because other children have decided to do something else |  |  |  |  |  |
| 11 | Have you ever had a confrontation with another child without getting into a fight? |  |  |  |  |  |
| 12 | You have been unable to borrow something you wanted |  |  |  |  |  |
| 13 | You have been told to calm down, to stay still |  |  |  |  |  |

Appendix 2. ***Daily report on parent-child conflicts***

Date:

Child's name:

Please circle who responds to the following items: father mother

| **WHEN WAKING UP** | | **Check**  **X** | | **IN PERSONAL HYGIENE** | **Check**  **X** |
| --- | --- | --- | --- | --- | --- |
| 1. S/he does not want to get up | |  | | 7. S/he does not cooperate in getting washed up (slow, distracted or playing) |  |
| 1. S/he appears angry about having to get up | |  | | 8. S/he appears cranky and demanding |  |
| 1. S/he attacks or insults because s/he does not want to get up | |  | | 9. S/he appears angry, but grooms himself/herself |  |
| 1. S/he appears cranky and demanding | |  | | 10. S/he attacks or insults because s/he does not want to get up |  |
| 1. S/he gets up well | |  | | 11. S/he grooms himself/herself correctly |  |
| 1. Another problem: | |  | | 12. Another problem: |  |
| **WHEN GETTING DRESSED** | | **Check**  **X** | | **AT BREAKFAST** | **Check**  **X** |
| 13. S/he does not cooperate when getting dressed (slow, distracted, or playful) | |  | | 19. S/he does not cooperate at breakfast (slow, distracted, or playful) |  |
| 14. S/he appears cranky and demanding | |  | | 20. S/he appears angry, but eats breakfast |  |
| 15. S/he appears angry, but gets dressed | |  | | 21. S/he appears cranky and demanding |  |
| 16. S/he attacks or insults because s/he does not want to get dressed | |  | | 22. S/he attacks or insults because he doesn't want to eat breakfast |  |
| 17. S/he gets dressed properly | |  | | 23. S/he does not eat breakfast |  |
| 18. Another problem: | |  | | 24. S/he eats breakfast |  |
|  | |  | | 25. Another problem: |  |
| **WHEN LEAVING THE HOUSE** | | **Check**  **X** | | **ON THE WAY HOME** | **Check**  **X** |
| 26. S/he appears sad | |  | | 34. S/he appears sad |  |
| 27. S/he appears angry | |  | | 35. S/he appears angry |  |
| 28. S/he is uncooperative (slow, distracted, or playful) | |  | | 36. She does not cooperate in collection (slow, distracted, playful) |  |
| 29. S/he appears cranky and demanding | |  | | 37. S/he appears cranky and demanding |  |
| 30. S/he attacks or insults because: | |  | | 38. S/he attacks or insults for a reason |  |
| 31. S/he disobeys the following: | |  | | 39. S/he disobeys something |  |
| 32. S/he gets out without a fuss | |  | | 40. S/he behaves well on the way |  |
| 33. Another problem: | |  | | 41. Another problem: |  |
| **AT LUNCH** | | **Check**  **X** | |  | **Check**  **X** |
| 42. S/he appears sad at lunch because of something | |  | | **IN THE AFTERNOON** |  |
| 43. S/he appears angry about something | |  | | 52. S/he appears sad about something |  |
| 44. S/he is uncooperative (slow, distracted, or playful) | |  | | 53. S/he appears angry about something |  |
| 45. S/he attacks or insults over food | |  | | 54. S/he attacks or insults for a reason |  |
| 46. S/he appears cranky and demanding | |  | | 55. S/he throws tantrums |  |
| 47. S/he disobeys (gets up from the chair, takes everything from the table...) | |  | | 56. S/he appears cranky and demanding |  |
| 48. S/he throws tantrums over food | |  | | 57. S/he disobeys in a particular situation |  |
| 49. S/he does not want to eat the food | |  | | 58. S/he fights with friends and/or siblings |  |
| 50. S/he eats food without a fuss | |  | | 59. S/he does not want to do homework |  |
| 51. Another problem: | |  | | 60. S/he does not want to take care of their personal hygiene (washing hands, brushing teeth...) |  |
|  | |  | | 61. S/he does not pick up toys |  |
|  | |  | | 62. S/he behaves well |  |
|  | |  | | 63. Another problem: |  |
| **AT DINNER** | | **Check**  **X** | | **AT BEDTIME** | **Check**  **X** |
| 64. S/he appears sad about something | |  | | 75. S/he appears sad |  |
| 65. S/he appears angry about something | |  | | 76. S/he appears angry because s/he does not want to go to sleep |  |
| 66. S/he is uncooperative at dinner (slow, distracted, playful) | |  | | 77. S/he is uncooperative when going to bed (slow, distracted, playful) |  |
| 67. S/he attacks or insults | |  | | 78. S/he appears cranky and demanding |  |
| 68. S/he throws tantrums | |  | | 79. S/he throws tantrums in order not to go to sleep |  |
| 69. S/he disobeys something | |  | | 80. S/he attacks or insults in order not to go to sleep |  |
| 70. S/he appears cranky and demanding | |  | | 81. S/he refuses to do bedtime chores |  |
| 71. S/he does not want to have dinner because s/he wants to do something else | |  | | 82. S/he goes to sleep without a fuss |  |
| 72. S/he does not want to eat their meal | |  | | 83. Another problem: |  |
| 73. S/he eats their meal without a fuss | |  | |  |  |
| 74. Another problem: | |  | |  |  |
|  |  | |  |  |  |
|  |  | |  |  |  |
